# Liquid-Crystal-Enabled Active Plasmonics: A Review

**DOI:** 10.3390/ma7021296

**Published:** 2014-02-18

**Authors:** Guangyuan Si, Yanhui Zhao, Eunice Sok Ping Leong, Yan Jun Liu

**Affiliations:** 1 College of Information Science and Engineering, Northeastern University, Shenyang 110004, Liaoning, China; E-Mail: siguang0323@hotmail.com; 2 Department of Engineering Science and Mechanics, The Pennsylvania State University, University Park, PA 16802, USA; E-Mail: yzz127@psu.edu; 3 Institute of Materials Research and Engineering, 3 Research Link, Singapore 117602, Singapore; E-Mail: leonge@imre.a-star.edu.sg

**Keywords:** liquid crystal, plasmonics, nanostructure, actively tunable device

## Abstract

Liquid crystals are a promising candidate for development of active plasmonics due to their large birefringence, low driving threshold, and versatile driving methods. We review recent progress on the interdisciplinary research field of liquid crystal based plasmonics. The research scope of this field is to build the next generation of reconfigurable plasmonic devices by combining liquid crystals with plasmonic nanostructures. Various active plasmonic devices, such as switches, modulators, color filters, absorbers, have been demonstrated. This review is structured to cover active plasmonic devices from two aspects: functionalities and driven methods. We hope this review would provide basic knowledge for a new researcher to get familiar with the field, and serve as a reference for experienced researchers to keep up the current research trends.

## 1. Introduction

Plasmonics [1–13], the study of the interaction between electromagnetic field and free electrons in a metal, has drawn increasing attention recently due to its huge potential for solving many eminent issues encountered by our world. Up to now, exciting plasmonic applications, for instance, super-resolution imaging [14–19], optical cloaking [20–23], and energy harvesting [24–29]*,* have been reported. Many other potential applications are under development. All these developments are attributed to the advanced nanofabrication techniques. Top-down nanofabrication techniques such as electron beam lithography [30–33] and focus ion beam milling [34–40] allow the accurate fabrication of structures at nanoscale with desirable trade-off on the high equipment expenses, as well as the considerably long time during sample preparation. Bottom-up techniques like self-assembly [41–43] can easily achieve regular patterns at a large scale at a rather low cost, but are not preferred for the cases that require accurate positioning and alignment with nanometer precision. Despite their strengths and weaknesses, both top-down and bottom-up techniques make their unique contributions to plasmonics by providing various nanostructures for plasmonic applications for different purposes. One major drawback of such plasmonic devices is that the fabricated nanostructures serve as passive devices providing consistent outputs given the same inputs, which greatly limits their application because of the additional investments required in fabricating another similar device with little change in the sample design. Thus, the new research field of active plasmonics emerges, which deals with reconfigurable function after the devices are fabricated, with the help of active mediums responsive to certain stimulus. Many active mediums have been used to build active plasmonic devices, including liquid crystals [44–59], molecular machines [60,61], elastic polymers [62–64], and chemical oxidation/reduction [65–67]. Among all the mentioned active mediums, liquid crystal stands out from all the rest because of its large birefringence on refractive index, low threshold on transition among different states, and versatile driven methods to cause the transitions. As a truly unique gift from nature, liquid crystals possess the smallest elastic constants and the largest birefringence among all known materials. In addition, their large birefringence spans the entire visible-infrared spectrum and beyond, which was first reported by Wu [68] in 1986. By virtue of their organic nature, they can be chemically synthesized and processed on a very large scale; they are also compatible with almost all technologically important optoelectronic materials. The alignment of liquid crystals can be easily controlled by many means, such as electricity, light, and acoustic waves, thus making them an excellent candidate for the development of active nanophotonics.

By integrating liquid crystals with plasmonic nanostructures, active plasmonic materials and devices with enhanced performance have been demonstrated. In this review, we summarize the recent research progress and achievements in liquid crystal based plasmonics. We hope the contents covered in this review can serve as a tutorial introduction to readers with little background in either plasmonics or liquid crystals. We also hope experienced researchers can further expand their knowledge of the given topic and be inspired to take this field to new horizons.

## 2. Plasmonics and Liquid Crystal Basics

### 2.1. Surface Plasmons

**Figure 1.**
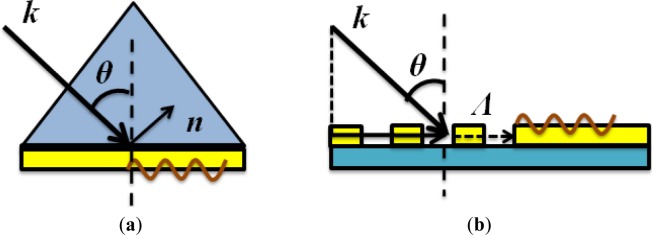
(**a**) Kretschmann configuration for surface plasmon excitation. The matching of wave vectors between surface plasmons and the incident light is achieved with the help of a prism with high refractive index and (**b**) the large surface plasmon wave vectors can also be obtained with the help of a grating. The grating provides additional wave vector components that can assist the conversion from incident light into surface plasmon waves.

Surface plasmons are a special kind of light formed by collective electron oscillations at the interface of a noble metal and dielectric. They can only propagate at the metal/dielectric interface and decay exponentially away from the interface. Surface plasmons exhibit different properties as their counterparts with the same frequencies, like much larger wave vectors, very short wavelengths, and limited propagating length with the dumping loss while propagating inside the metal. They also fit into Maxwell equations, and the wave vector supported by different combinations of metal and dielectric interfaces can be calculated by solving the equation as shown below:

$$k_{\text{sp}}=k_{0}\sqrt{\frac{\varepsilon_{\text{m}}\varepsilon_{\text{d}}}{\varepsilon_{\text{m}}+\varepsilon_{\text{d}}}}$$ (1)

where *k*_sp_ represents the wave vector of surface plasmons and *k*_0_ is the wave vector of the incident light in vacuum. ε_m_ and ε_d_ are dielectric constants of metal and dielectric, respectively. From the equation above, it is clear that direct conversion from propagating electromagnetic waves into surface plasmons is impossible due to the mismatch of their wave vectors. There are many different ways to solve this problem and the most commonly used ones are Kretschmann/Otto configurations and gratings, as illustrated in Figure 1.

The Kretschmann configuration consists of a prism with high refractive index attaching to a glass slide with refractive index matching to oil; the other side of the glass slide is covered with a thin layer of gold film. There are two metal/dielectric interfaces found in this system as air/gold and glass/gold interfaces. According to Equation (1), the two different interfaces support different surface plasmon modes. Incident light from the prism side cannot excite surface plasmons on both interfaces; however, with the help of a large refractive index from the prism side, it is possible that the leakage horizontal wave vectors from the incident light on the prism side will match the surface plasmon wave vector on the air side, validating the relation of:

$$n_{\text{p}}k_{0}\text{sin}\theta =k_{0}\sqrt{\frac{\varepsilon_{\text{m}}\varepsilon_{\text{air}}}{\varepsilon_{\text{m}}+\varepsilon_{\text{air}}}=k_{\text{sp},\text{air}}}$$ (2)

where *n*_p_ is the refractive index of the prism; ε_air_ is the dielectric constant of air and θ represents the incident angle from the prism side. One alternative way is to use gratings, where the grating constant provides the missing momentum components from the incident light to surface plasmons. In order to do so, the period of the gratings should be at the close range of the incident wavelength. The equation describing this process can be expressed as:

$$k_{\text{sp}}=n_{i}k_{0}\text{sin}\theta \pm m\Lambda$$ (3)

Λ*=*2π/*d* is the grating constant (*d* is the period of grating). *n_i_* is the refractive index of incident plane; θ is the incident angle and *m* is integer. Both methods are well investigated and have found their wide applications in plasmonic-related research with many other excitation methods being developed. Sometimes they can be combined together to maximize the system performance. The excitation conditions have to be strictly followed to allow surface plasmons to be excited; while a less strict method requiring irregular sharp edges can also excite surface plasmons through the strong scattering caused by the wave vector match; however, the exciting efficiency is quite low since only a small amount of wave vectors can match with surface plasmon wave vectors during the exciting process.

Plasmonics is the further extension of surface plasmons, with its focus on the light-matter interaction occurring at nanoscales along with different nanoparticles and nanostructures. At this scale, light can not only be confined at the metal/dielectric interface, but also can be squeezed, tightly focused/confined, and manipulated at will with the assistance from various nanostructures. Its huge potential in various applications such as energy, biomedicine, metamaterials and photonic/quantum computing has been explored and revealed from time to time.

### 2.2. Liquid Crystals

Liquid crystals have attracted a lot of attention due to the unique electro-optical and thermo-optical properties and have been used in numerous applications [69,70], such as liquid crystal displays for televisions, projectors, watches, cellphones, spatial light modulators for adaptive optics in real time optical imaging, optical switches and attenuators for telecommunications, and optical phase arrays for beam steering, *etc*. The research on liquid crystal materials and devices is of special importance not only to the quality of our daily life but also to the development of the human society.

**Figure 2.**
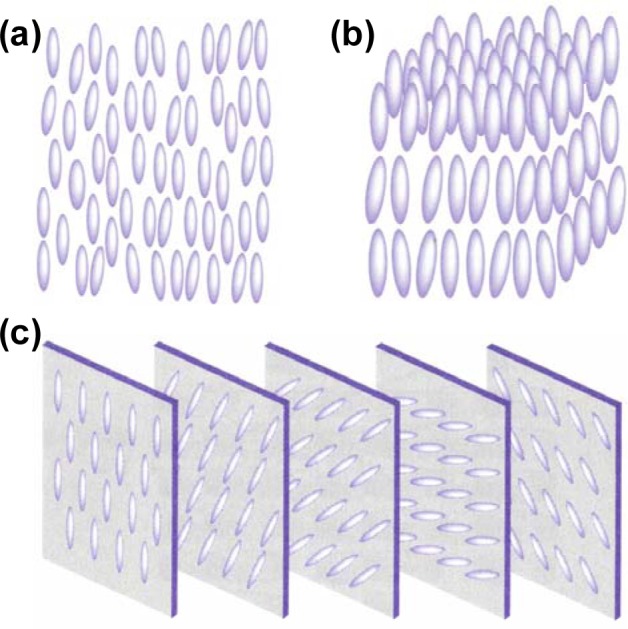
Phases of liquid crystals: (**a**) nematic phase; (**b**) smectic phase and (**c**) cholesteric phase.

Generally speaking, there are three phases of thermotropic liquid crystals, known as the nematic phase, smectic phase, and cholesteric phase. Figure 2a illustrates the nematic phase in which only long range orientational order of the molecular axis exists. Figure 2b shows the smectic phase in which one-dimensional translational order as well as orientational order exists. Figure 2c illustrates the cholesteric phase of a liquid crystal by viewing the distribution of molecules at several planes that are perpendicular to the helical axis. The cholesteric phase is actually a nematic type of liquid crystal except that it is composed of chiral molecules. As a consequence, the structure acquires a spontaneous twist in line with a helical axis normal to the director. The twist can be right-handed or left-handed depending on the molecular chirality. Under proper treatment, a slab of nematic liquid crystal can be obtained with a uniform alignment of the director. Such a sample exhibits uniaxial optical symmetry with two principal refractive indices *n*_o_ and *n*_e_. The ordinary refractive index *n*_o_ is for light with electric field polarization perpendicular to the director and the extraordinary refractive index *n*_e_ is for light with electric field polarization parallel to the director. The birefringence (or optical anisotropy) is defined as *∆n = n*_e_ *− n*_o_. If *n*_o_ < *n*_e_, liquid crystal is of positive birefringence, whereas if *n*_o_ > *n*_e_, it is of negative birefringence. In classical dielectric theory, the macroscopic refractive index is related to the molecular polarizability at optical frequencies. The existence of the optical anisotropy is mainly due to the anisotropic molecular structures. In the optical regime, $\varepsilon_{∥}=\varepsilon_{0}n_{e}^{2}$ and $\varepsilon_{⊥}=\varepsilon_{\text{0}}n_{0}^{2}$, thus $\Delta \varepsilon =\varepsilon_{0}(n_{\text{e}}^{2}-n_{0}^{2})$ For most liquid crystals, the ordinary refractive index is around 1.5. The optical anisotropy plays an essential role in changing the polarization state of light in liquid crystals.

**Figure 3.**
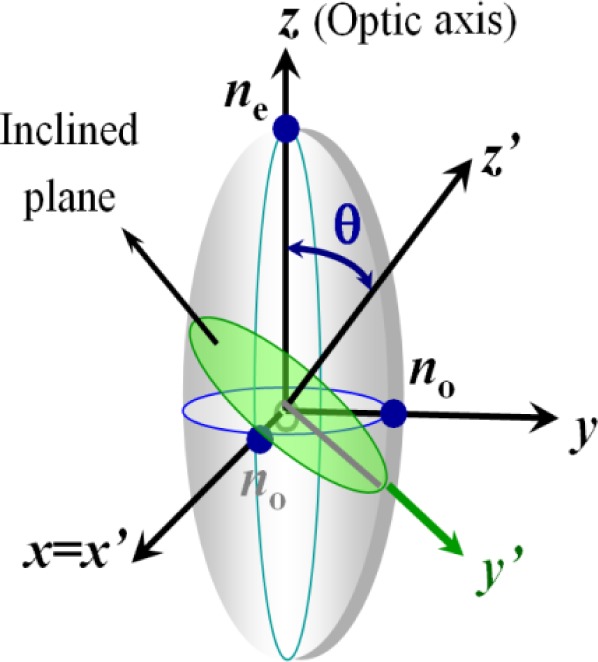
Light propagation in uniaxial mediums.

For light propagating in the liquid crystal with an incident angle θ relative to the optical axis, shown as Figure 3, two eigen refractive indices are seen by the light with two modes of propagation. One mode with its polarization direction perpendicular to the plane formed by the wave vector and the optic axis is called ordinary wave, which sees a refractive index of *n*_o_ (independent of the incident angle ). The other one with its polarization direction parallel to the plane formed by the wave vector and the optic axis is called extraordinary wave, which sees an effective refractive index (dependent of the incident angle θ). The effective refractive index seen by the light can be written as:

$$n_{\text{eff}}(\theta )={\left[\frac{{\text{cos}}^{2}\theta }{n_{0}^{2}}+\frac{{\text{sin}}^{2}\theta }{n_{\text{e}}^{2}}\right]}^{-\frac{1}{2}}$$ (4)

**Figure 4.**
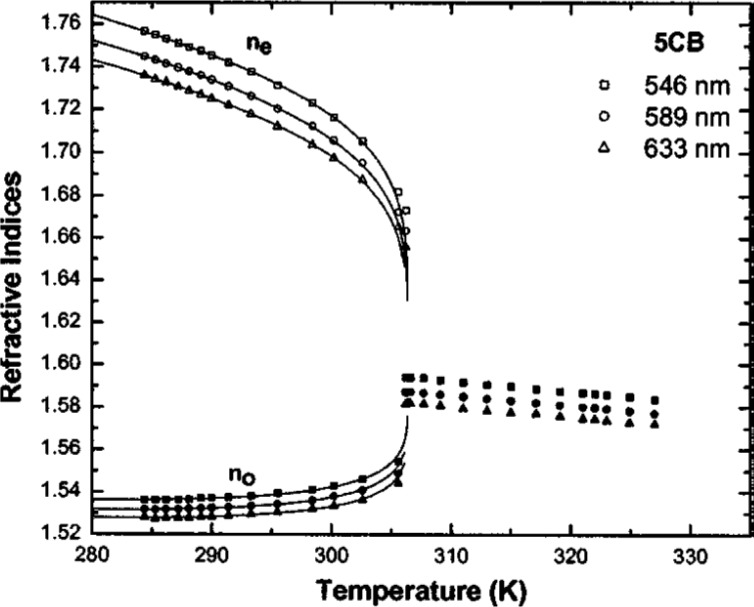
Temperature-dependent refractive indices of 5CB at λ = 546, 589 and 633 nm. Squares, circles, and triangles are experimental data for refractive indices measured at λ = 546, 589 and 633 nm, respectively. Figure 4 is adapted from reference [71].

Similarly, the optical anisotropy is also a function of temperature and approximately linearly proportional to the order parameter *S*. A general trend of the refractive index is plotted in Figure 4 as a function of temperature [71]. As we can see, the optical anisotropy decreases as the temperature increases and vanishes in the isotropic phase.

By combining liquid crystals with plasmonics, a new research category of liquid crystal based plasmonics as a branch of active plasmonics is eminent and starts to draw increasing attention from researchers all over the world. Here, we summarize recent research progresses and achievements of the liquid crystal plasmonics to share our thoughts about the field, and hopefully inspire talented researchers who are interested in pursuing excellence in this research area.

## 3. Active Plasmonic Devices Based on Liquid Crystals

The birefringence of liquid crystal is demonstrated as a difference in refractive index, making it an ideal active medium for active plasmonic devices for different applications, such as plasmonic switches [50–54], active plasmonic color filters [30,35,39] and plasmonic waveguides [72]. While the concept of applying liquid crystal to plasmonic nanostructure is straightforward, there are various ways to pump or drive the liquid crystal to achieve a noticeable refractive index change. In the following, representative liquid crystal based active plasmonic devices will be categorized and discussed according to the driving methods.

### 3.1. Electric-Field-Driven Method

**Figure 5.**
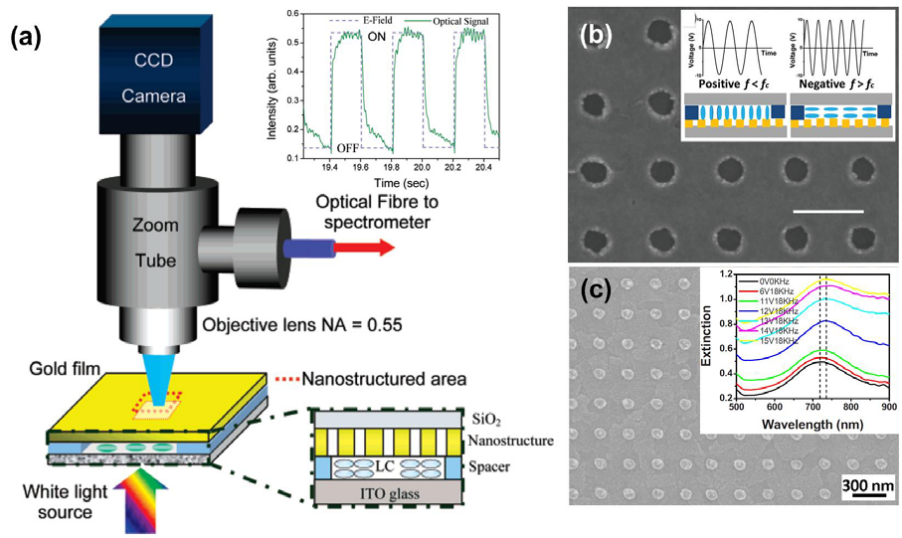
(**a**) Schematic and experimental setup of a plasmonic switch consisting of nanostructures and liquid crystals. Electric field is generated by applying voltages across the gold film and bottom ITO glass, switching effect at the wavelength of 670 nm is shown in the inset; (**b**) SEM image of nanohole array and the working mechanism of DFLC. The scale bar indicates one period length of 300 nm; (**c**) SEM image of gold nanorods of another design of plasmonic switch using DFLC. The inset extinction spectrum shows the plasmonic resonance change under different voltage and frequency combinations. Figure 5a–c is adapted from reference [55], reference [47] and reference [50], respectively.

Electric fields have very strong impacts on rod shape of liquid crystal molecules. When the liquid crystal molecules are subject to an electric field, one end of a molecule has positive charges, while the other end is negatively charged, forming an electric dipole. As a result, the director of the liquid crystal molecules will be re-oriented along the direction of an external electric field. Therefore, electric field is the most commonly used method to drive the liquid crystal devices. Utilizing this characteristic of the liquid crystal, efforts have been made to combine liquid crystals with periodically nanostructured metal films to develop electronically controlled transmission, reflection, and absorption of the plasmonic structures, concerning their applications on switches, filters, and modulators. One example is given by Dickson *et al*. [55], who have demonstrated precise control over surface plasmon dispersion and transmission of gold nanohole arrays using liquid crystals. A schematic of the experimental setup is shown in Figure 5a. A liquid crystal layer with the thickness controlled by the spacers is sandwiched between a conductive indium-tin-oxide (ITO) glass substrate and a gold film with nanohole arrays milled by using a focused ion beam. White light impinging from the ITO side of the sample will pass through the liquid crystal layer and then reach the nanostructures to excite different surface plasmon polariton modes. Upon applying voltages, electric fields are built up across the ITO substrate and the gold film, and the liquid crystal molecules tend to realign along the electric field direction. As a result, effective refractive index at the interface of gold/liquid crystal is changed and subsequently results in a variation on the surface plasmon dispersion relation; thus, changing the excitation conditions of certain plasmon modes. This change is phenomenally reflected as a transmission modulation from the spectrum and provides us an active control of surface plasmon modes. De Sio *et al.* [57] also reported that a carrier accumulation layer in the proximity of the ITO substrate can be formed under the external electric field, hence modifying the effective refractive index around the metallic nanostructures and affecting their plasmonic resonances subsequently. To achieve an additional degree of control freedom, dual-frequency liquid crystal (DFLC) therefore is much preferred in designing an active plasmonic device, because it responds to the applied voltages with different frequencies. DFLC can change its sign of dielectric anisotropy from positive to negative, or negative to positive, upon the frequency change of the applied electric fields [73,74]. Complementary gold nanodisk and nanohole arrays have been demonstrated using electron-beam lithography followed by lift-off process, which are promising carriers to build plasmonic devices, as shown in Figures 5b,c. By overlaying a DFLC layer on these nanodisk and nanohole structures, reversible tuning of plasmonic resonances and transmission have been demonstrated. In these hybrid systems, homeotropic alignment of DFLC was set at the initial state by using a self-assembly alignment layer of hexadecyl trimethyl ammonium bromide (HTAB). When the frequency of the applied electric field is lower than the crossover frequency of DFLC, and all the liquid crystal molecules will keep the homeotropic alignment, *i.e.*, perpendicular to the substrate, which is independent of applied voltages. Once the applied frequency exceeds the crossover frequency, the liquid crystal molecules tend to prefer the homogeneous alignment, *i.e.*, parallel to the substrate, which is dependent on applied voltages. As a result, the switching/tuning effect with various transmissions can be easily obtained by varying applied voltages. Another example of tuning nanorod plasmons using liquid crystal is given by Khatua and co-workers [58]. With the applied external voltage as low as 4 V, complete modulation of the polarized scattering intensities of individual gold nanorods can be simply achieved. Note that the demonstrated strategy can be readily translated to other antenna architectures with more complex designs and various plasmonic elements for the electrical manipulation of light in structures with nanoscale dimensions.

**Figure 6.**
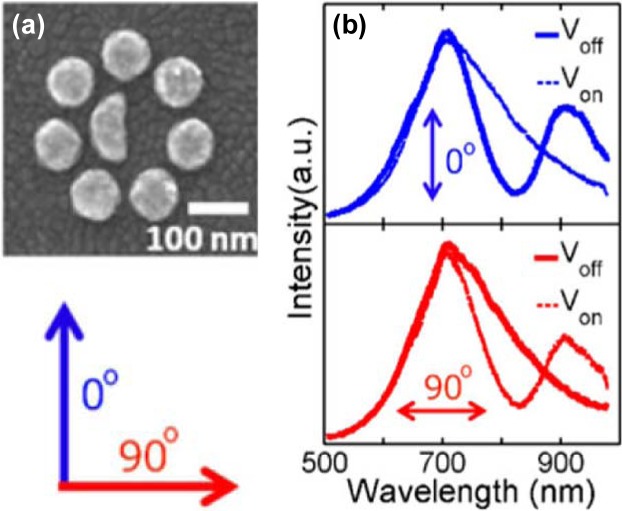
(**a**) SEM image of the octamer structure (top), which shows Fano-like and non-Fano like spectra with polarization at 0° and 90° (bottom); (**b**) the scattering spectra of the octamer structure in the V_on_ and V_off_ states measured at a detection: the homogenous nematic phase in the V_off_ state (top) and the twisted nematic phase in the V_on_ state (bottom). All figures are adapted from reference [75].

Recently, a plasmonic Fano switch has also been demonstrated with a more specific design [75]. The device consists of a specifically designed cluster of gold nanoparticles fabricated using electron-beam lithography. The cluster comprises a large hemi-circular disc surrounded by seven smaller nanodiscs as shown in Figure 6a. Interactions between LSPRs of the individual nanoparticles within a cluster lead to a so-called Fano resonance, which is a result of near-field coupling between collective “bright” and “dark” plasmon modes of the cluster. By breaking the symmetry of the nanoparticle cluster through the hemi-circular centre disc, the Fano resonance is polarization-dependent and can only be observed for one polarization of the incident light. As a result, no Fano resonance appears in the light spectrum, for incident light that is polarized at 90° to this direction. The nanoparticle clusters are incorporated into liquid crystals in which the molecules at the device interface can be rotated in plane by 90° when an AC voltage of about 6 V is applied. The field creates a twist in the overall alignment direction of the crystals, which leads to a phase transition from “homogenous nematic” (voltage off) to “twisted nematic” (voltage on). Due to the birefringence of the liquid crystal, the voltage-induced phase transition causes an orthogonal rotation of the scattered light from the plasmonic clusters as it travels through the device. This results in switching between the optical response with and without the Fano resonance, as shown in Figure 6b.

### 3.2. Light-Driven Method

**Figure 7.**
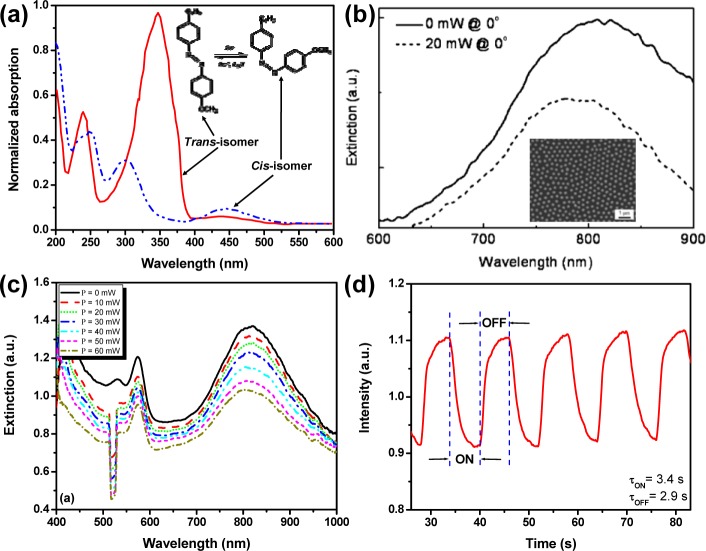
(**a**) The spectral changes of BMAB under UV exposure that reflects its trans-cis isomerization process; (**b**) extinction spectra (normal incidence) of a photoresponsive liquid crystal/gold nanodisk array cell before (solid curve) and after (dashed curve) the application of a 20 mW pump light (λ = 420 nm) at an incident angle of 45°. Thirty nm blue-shift of the extinction peak can be observed. The alignment of liquid crystal molecules is achieved mechanically using photo-responsive azobenzene. (**c**) The spectral changes of extinction in another azo dye (methyl red) doped grating integrated with the same gold nanodisk array and (**d**) switching effect and time of holographic polymer-dispersed liquid crystals gratings upon laser excitation. Figure 7a,b is adapted from references [39,52], respectively. Figure 7c,d is adapted from reference [83].

All-optical tuning method has been widely applied in liquid crystal based optical elements like spatial light modulator, filter, reflector, *etc*.; and it has many advantages such as non-contact tuning, low power consumption, and friendly integration, making it an exciting concept to be applied on light driven liquid crystal based devices. As compared to the electrical tuning method, light driven method requires: (1) no conductive ITO substrates; (2) low power consumptions and (3) large operation windows covering UV to mid-IR. Azobenzene and its derivatives are a widely used guest in a liquid crystal host. They have a trans-cis reversible isomerization dynamic behavior upon exposure to a UV or visible light beam. The isomerization will disrupt the local order of surrounding liquid crystal molecules in the mixture, resulting in realignment of those liquid crystal molecules to exhibit a refractive index change. Such an index change has been utilized for dynamic control of many photonic devices, such as switchable gratings [51,76–79] and photonic crystals [80,81]. It is also straightforward for dynamic control of surface plasmons [52,82,83]. One example of a light-driven plasmonic switch has been demonstrated by Hsiao *et al*. [52] with a light responsive liquid mixture consisting of nematic liquid crystals and an azobenzene derivative. 4-butyl-4’-methyl-azobenzene (BMAB) is used to induce the switching effect. Figure 7a shows the spectral changes of BMAB under UV exposure that reflects its trans-cis isomerization process. Figure 7b shows the extinction spectra of a photoresponsive liquid crystal/gold nanodisk array (see the inset of Figure 7b) cell at normal incidence of a probing beam before (solid curve) and after (dashed curve) the light pump (λ = 420 nm, I = 20 mW) at the incident angle of 45°. One can observe a 30 nm blue-shift of the extinction peak. Figure 7c shows the spectral changes of extinction in another azo dye (methyl red) doped grating integrated with the same gold nanodisk array [83]. The switching effect before and after the light pump is shown in Figure 7d with both switching “ON” and “OFF” time less than 4 seconds, which is the typical response time for the azobenzene and its derivatives under the continuous light pump. However, under the pulsed laser pump, the response time of azobenzene doped liquid crystals could reach nanoseconds since the photoisomerization of azobenzene from trans-state to cis-state could undergo the pathway of either π−π* rotation or n−π* inversion with different response speeds [84].

**Figure 8.**
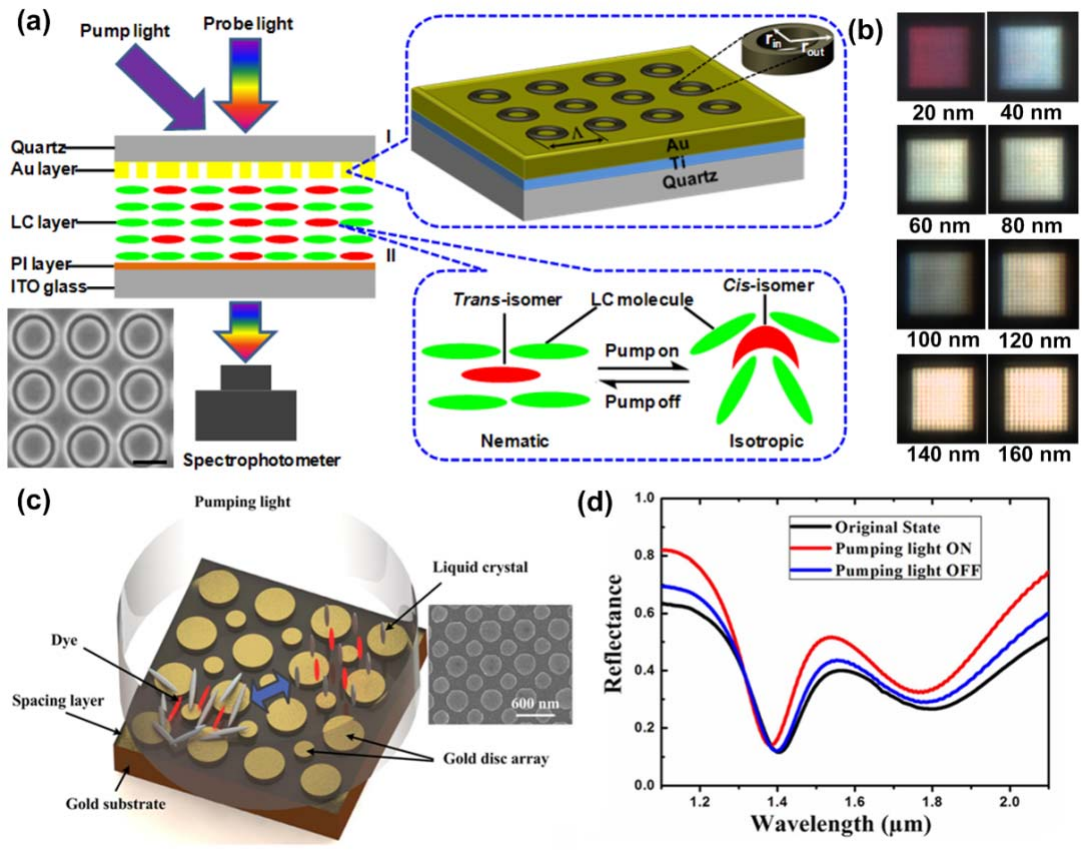
(**a**) Light driven plasmonic color filter tuned by liquid crystals. The nanostructure used is a new type AAA that can generate different colors by changing the sizes and periods of AAA. One example of AAA is given in the inset; (**b**) different colors generated using plasmonic color filters. By pumping the liquid crystal mixtures to realign the liquid crystal molecule orientation, intensity modulation on the ultra-small color filter can be achieved and demonstrated in the reported work.; (**c**) one representative case of reconfigurable plasmonic absorber using liquid crystal. The small and big nanodisk arrays in the inset are designed to produce two absorption maximums to improve the performance of the plasmonic absorbers and (**d**) light sensitive liquid crystal mixture is used to tune the absorption dips in real time. Experimental results confirm a tuning range around 25 nm in the near infrared range. Figure 8a,b is adapted from reference [39] while Figure 8c,d is adapted from reference [45].

Based on the same working mechanism, we have also demonstrated light-driven plasmonic color filters with high optical transmission and narrow bandwidth by overlaying a layer of photoresponsive liquid crystals on gold annular aperture arrays (AAAs). The schematic of the light-driven plasmonic color filters and experimental setup is shown in Figure 8a. The enlarged Part I in Figure 8a shows the fabricated square pattern of gold AAAs using focused ion beam lithography. The inner and outer radii of each individual aperture are labeled as *r*_in_ and *r*_out_, respectively. The magnified Part II in Figure 8a shows the working mechanism of the optical driving process: a reversible nematic–isotropic phase transition induced by the trans-cis photoisomerization of the photochromic liquid crystals. The gold AAAs with various aperture sizes and periods have been reported to generate different colors in the visible range. The addition of photoreponsive liquid crystals will then make the transmission of color filters optically tunable. The photosensitive liquid crystal mixture has similar composition as other mixtures discussed above. With its large birefringence Δ*n =* 0.225 (at the wavelength of 589 nm, *n*_o_ = 1.521, *n*_e_ = 1.746), it offers major intensity modulation of the transmitted color through the plasmonic color filter shown in Figure 8b, where the intensity of each individual color generated from plasmonic structures can be further tuned, as confirmed by both simulations and experiments in the reported work [39]. Therefore, any color could be achieved through the composition of three tunable primary red, green and blue color filters. This all-optical tuning behavior is highly reversible and reproducible, making such a kind of color filter promising in all-optical information processing and displays. Another useful device is plasmonic absorbers, which can exhibit extraordinary absorption efficiency of more than 90% at designated wavelength bands by engineering nanostructures with different shapes and sizes. Inclusion of birefringent nematic liquid crystals in their makeup can make the absorption bands electrically or optically tuned or modulated, hence providing additional freedom to control the absorption bands. Zhao *et al*. have experimentally demonstrated a light-driven plasmonic absorber based on a nematic liquid crystal host doped with an azo dye [45]. Figure 8c shows the schematic of light-driven reconfigurable plasmonic absorber. A cladding layer of liquid crystals mixed with azo dyes is added on top of the plasmonic absorber. The use of azo dye is to effectively absorb the light energy and transfer the absorbed energy to heat up the liquid crystal environment. The liquid crystal used here is thermo-sensitive type with transition temperature of 35 degrees (5CB). Liquid crystal will transform from a nematic to isotropic state after heating up, along with a gradual refractive index change from 1.61 to 1.56 with the increase of temperature. The refractive index change modifies the frequency selected surfaces as well as the resonance frequency of the resonant cells formed by top gold nanostructures, and a bottom gold layer. A noticeable shift of the absorption dips of around 25 nm has been confirmed in Figure 8d. Note that the photosensitive liquid crystals with an azo group in their mesogenic structure exhibit a higher solubility compared to non-mesogenic azo-dyes [85–87]. Furthermore, the orientational order of liquid crystals still remains high in the presence of mesogenic azo dopants and their photoisomerization effect on the host liquid crystal is stronger than that of non-mesogenic dopants.

### 3.3. Surface Acoustic Wave (SAW)-Driven Method

**Figure 9.**
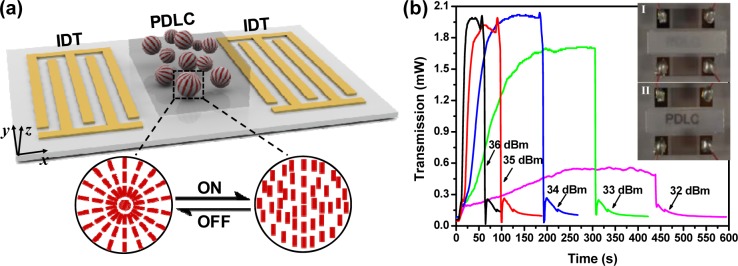
(**a**) Light shutter based on acoustic modulation of PDLC. The shutter effect is caused by realignment of liquid crystal molecules; (**b**) experimental demonstration of acoustic driven light shutter. Letters of “PDLC” beneath the PDLC film can be clearly observed after SAW applied. Transmission changes before and after SAW applied is shown. Response time can be estimated. All figures are adapted from reference [49].

Although liquid crystal driven by surface acoustic waves (SAWs) is loosely connected to the scope of this review paper, we put it here as an insight into prospective integration of liquid crystal with micro- and nano-technologies. SAWs are sound waves propagating along the surface of a piezoelectric substrate. Its low power consumption and intactness from the driven medium make it an effective approach to re-align the liquid crystal molecules. Liu *et al*. [49] have demonstrated a SAW-driven light shutter based on polymer-dispersed-liquid-crystal (PDLC). A schematic of the designed light shutter is shown in Figure 9a. It consists of a PDLC film layer and two inter-digital transducers (IDTs) on a piezoelectric substrate. The PDLC film consists of liquid crystal droplets randomly distributed in a polymer matrix. Before applying SAW, the PDLC film exhibits strong scattering due to the refractive index mismatch between the polymer matrix and liquid crystal droplets, thus demonstrating a non-transparent state. When a radio frequency signal is applied to IDT, SAW is excited and then propagates along the surface. When the propagating SAW encounters the PDLCs, a longitudinal wave is induced and leaks into the PDLCs. The longitudinal wave will cause the liquid crystal molecules to realign to eliminate the refractive index mismatch, *i.e.*, their ordinary refractive index matches with that of the polymer. As a result, the PDLC film becomes completely transparent. The acoustic wave induced streaming inside the liquid crystal rich regions as well as the temperature change caused by the attenuation of this longitudinal wave are believed to be key factors to reorient the alignment of liquid crystal molecules. Figure 9b shows the experimental results marking the “ON” and “OFF” states of the SAW-driven shutter. The word “PDLC” can be clearly observed by turning ‘ON’ the shutter, and the film is opaque when the shutter is “OFF”. We believe that such a SAW-driven liquid crystal mechanism will be applicable to the plasmonic devices as well. Our research on SAW-driven plasmonic devices incorporated liquid crystals is currently on-going.

### 3.4. Heat-Driven Method

**Figure 10.**
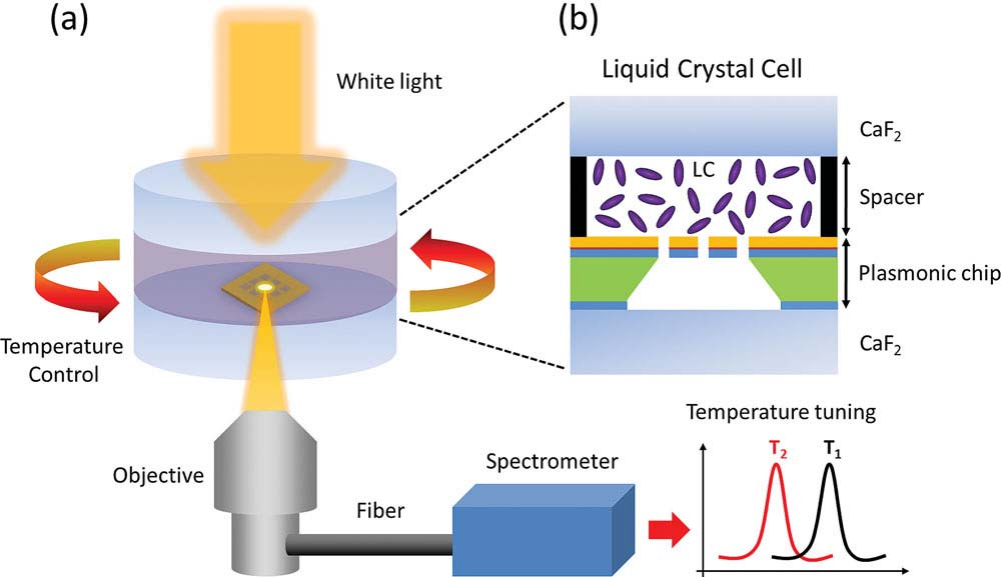
(**a**) Schematic view of the experimental setup containing a liquid crystal cell thermally controlled by a heat bath, white light incident source collected by an objective lens and a spectrometer collecting the plasmonic response for different temperature values and (**b**) zoomed schematic of the liquid crystal cell between the upper CaF_2_ window and the plasmonic chip. This figure is adapted from reference [90].

Temperature also plays an important role in affecting the liquid crystal refractive indices. As the temperature increases, the extraordinary refractive index, *n*_e_, behaves differently from the ordinary refractive index, *n*_o_. The derivative of *n*_e_ (*i.e.*, ∂*n*_e_/∂*T*) is always negative. However, ∂*n*_o_/∂*T* changes from negative to positive as the temperature exceeds the crossover temperature [88,89]. For many liquid crystals, the temperature-dependent refractive indices can be accurately controlled, hence providing another effective means to develop active plasmonics. As previously discussed in Figure 4, we can clearly observe that the optical anisotropy decreases as the temperature increases and finally disappears (*i.e.*, *n*_e_ = *n*_o_) at the isotropic phase regardless of wavelength. Based on this mechanism, Altug group has demonstrated the thermal tuning of SPPs using liquid crystals [90], as shown in Figure 10. Varying the temperature within the nematic phase from 15 to 33 °C, they demonstrated a refractive index change as large as ≈0.0317, thus enabling a tuning of plasmonic wavelength as large as ≈19 nm. The ability to control the order of liquid crystal molecules from nematic to isotropic phase provides an efficient way of spectral tuning. At the phase transition temperature, more than 12 nm shift has been achieved via changing the temperature by only ≈1 °C corresponding to a refractive index change of ≈0.02. It has been known that plasmonic nanoparticles can induce a photo-thermal heating effect, which depends on the geometries of the nanoparticles strongly and has been extensively investigated [91–94]. It is expected that with the involvement of liquid crystals, such a photo-thermal heating effect will also affect the effective refractive index of the liquid crystals and hence tune the plasmonic resonances as well.

## 4. Summary and Outlooks

In summary, we have reviewed the recent research progress and achievements in liquid crystal based plasmonics. Different driving methods of the liquid crystal based plasmonic devices have been categorized and discussed. The versatile driving methods of the liquid crystals provide enormous freedom in the design and implementation of the plasmonic devices. Although tremendous efforts have been focused on the development of liquid crystal based active plasmonic devices in recent years, many challenges still remain before they can be efficiently used in practice. In the below, we provide a more specific description of these challenges as well as future demands.

### 4.1. Current Challenges

Currently, most liquid crystal based plasmonic devices are fabricated by mechanically assembling liquid crystals on passive plasmonic nanostructures on a substrate. However, large-area uniform liquid crystal alignment on the nanostructures is still a big challenge given the fact that only the liquid crystal molecular layer that is near (generally < 100 nm) to the plasmonic nanostructures affects the plasmonic signals. In order to achieve large-area uniform liquid crystal alignment in plasmonic devices, one possible way is to co-self-assemble liquid crystals and plasmonic nanoparticles [95–98]. Based on the co-self-assembly method, uniform areas up to 100 μm^2^ have been achieved, which is promising for device development.

### 4.2. Future Demands

#### 4.2.1. Fast Response Liquid Crystals

High speed is always demanding for active plasmonic devices, especially for future development of nanophotonic circuitry/chips with multiple functions assembled. Currently, most liquid crystal based plasmonic devices have a millisecond scale response using the electric-field-driven method. To have a faster speed, one could choose some special liquid crystals, such as ferroelectric liquid crystals, which in general have microsecond response time. In confining the liquid crystal in a polymer matrix, it will also help increase the response speed. For example, millisecond response time has been achieved in a polymer-dispersed liquid crystal (PDLC) system, in which microscale liquid crystal droplets are randomly confined in a polymer matrix [99,100]. While in a holographic polymer-dispersed liquid crystal (HPDLC) system, in which nanoscale liquid crystal droplets are periodically confined in a polymer matrix, microsecond response time has been demonstrated [101–103]. More recently, polymer network liquid crystal (PNLC) [104] and polymer-stabilized blue phase liquid crystal (BPLC) [105] have received much attention since both systems can achieve sub-millisecond response time.

#### 4.2.2. Large Birefringence Liquid Crystals

Currently, liquid crystals employed in most reported work have the birefringence of 0.1–0.2. Although such a birefringence has already given detectable changes in terms of peak shift or intensity, there is much room for further improvement. To achieve more pronounced changes, liquid crystals possessing larger birefringence are highly desired. Thus far, liquid crystals with large birefringence of >0.4 [106–108] and even >0.7 [109] have been reported, respectively. We believe that much enhanced performance, such as large peak shift and intensity modulation, will be achieved by integrating the large birefringent liquid crystal into plasmonic nanostructures, hence beneficial to the development of active plasmonics.

#### 4.2.3. Multi-Function or Multi-Control Integration

At the current stage, a single function of liquid crystal based plasmonic devices has been demonstrated in terms of proof-of-concepts. Looking ahead, it will be great that a single device could do multiple functions. Liquid crystal enabled plasmonic devices look promising for achieving multiple functions. In addition, multi-mode controls of liquid crystals will also lend a convenient hand and allow us to choose their functions freely under certain circumstances. For example, azo-dye-doped liquid crystals or azobenzene liquid crystals can respond to not only electric fields but also light [110], which gives us more freedom to control them.

## References

[1] Barnes W.L., Dereux A., Ebbesen T.W. (2003). Surface plasmon subwavelength optics. Nature.

[2] Ebbesen T.W., Lezec H.J., Ghaemi H.F., Thio T., Wolff P.A. (1998). Extraordinary optical transmission through sub-wavelength hole arrays. Nature.

[3] Bozhevolnyi S.I. (2009). Plasmonic Nanoguides and Circuits.

[4] Degiron A., Smith D.R., Brongersma M.L., Kik P.G. (2007). Surface Plasmon Nanophotonics.

[5] Garcia-Vidal F.J., Martin-Moreno L., Pendry J.B. (2005). Surfaces with holes in them: New plasmonic metamaterials. J. Opt. A Pure Appl. Opt.

[6] Cai W., Shalaev V. (2010). Optical Metamaterials.

[7] Smith S.J., Purcell E.M. (1953). Visible light from localized surface charges moving across a grating. Phys. Rev.

[8] Veselago V.G. (1967). The electrodynamics of substances with simulataneous negative values of ε and μ. Usp. Fiz. Nauk.

[9] Zayats A.V., Smolyaninov I.I., Maradudin A.A. (2005). Nano-optics of surface plasmon-polaritons. Phys. Rep.

[10] Smolyaninova V.N., Smolyaninov I.I., Kildishev A.V., Shalaev V.M. (2010). Broadband transformation optics devices. Materials.

[11] Dutta N., Mirza I.O., Shi S., Prather D.W. (2010). Fabrication of large area fishnet optical metamaterial structures operational at near-IR wavelengths. Materials.

[12] Alu A., Engheta N. (2011). Emission enhancement in a plasmonic waveguide at cut-off. Materials.

[13] Si L., Jiang T., Chang K., Chen T., Lv X., Ran L., Xin H. (2011). Active microwave metamaterials incorporating ideal gain devices. Materials.

[14] Pendry J.B. (2000). Negative refraction makes a perfect lens. Phys. Rev. Lett.

[15] Fang N., Lee H., Sun C., Zhang X. (2005). Sub-diffraction-limited optical imaging with a silver superlens. Science.

[16] Pendry J.B., Smith D.R. (2006). The quest for the superlens. Sci. Am.

[17] Vincenti M.A., D’Orazio A., Cappeddu M.G., Akozbek N., Bloemer M.J., Scalora M. (2009). Semiconductor-based superlens for subwavelength resolution below the diffraction limit at extreme ultraviolet frequencies. J. Appl. Phys.

[18] Liu Z., Durant S., Lee H., Pikus Y., Fang N., Xiong Y., Sun C., Zhang X. (2007). Far-field optical superlens. Nano Lett.

[19] Liu Z., Durant S., Lee H., Pikus Y., Xiong Y., Sun C., Zhang X. (2007). Experimental studies of far-field superlens for sub-diffractional optical imaging. Opt. Express.

[20] Schurig D., Mock J.J., Justice B.J., Cummer S.A., Pendry J.B., Starr A.F., Smith D.R. (2006). Metamaterial electromagnetic cloak at microwave frequencies. Science.

[21] Pendry J.B., Schurig D., Smith D.R. (2006). Controlling electromagnetic fields. Science.

[22] Jacob Z., Narimanov E.E. (2008). Semiclassical description of non magnetic cloaking. Opt. Express.

[23] Smolyaninov I.I., Smolyaninova V.N., Kildishev A.V., Shalaev V.M. (2009). Anisotropic metamaterials emulated by tapered waveguides: application to optical cloaking. Phys. Rev. Lett.

[24] Zhu G., Lin Z., Jing Q., Bai P., Pan C., Yang Y., Zhou Y., Wang Z.L. (2013). Toward large-scale energy harvesting by a nanoparticle-enhanced triboelectric nanogenerator. Nano Lett.

[25] Walter M.J., Borys N.J., van Schooten K.J., Lupton J.M. (2008). Light-harvesting action spectroscopy of single conjugated polymer nanowires. Nano Lett.

[26] Aubry A., Lei D.Y., Fernandez-Dominguez A.I., Sonnefraud Y., Maier S.A., Pendry J.B. (2010). Plasmonic light-harvesting devices over the whole visible spectrum. Nano Lett.

[27] Andreussi O., Biancardi A., Corni S., Mennucci B. (2013). Plasmon-controlled light-harvesting: Design rules for biohybrid devices via multiscale modeling. Nano Lett.

[28] Kim I., Bender S.L., Hranisavljevic J., Utschig L.M., Huang L., Wiederrecht G.P., Tiede D.M. (2011). Metal nanoparticle plasmon-enhanced light-harvesting in a photosystem I thin film. Nano Lett.

[29] Dang X., Qi J., Klug M.T., Chen P., Yun D.S., Fang N.X., Hammond P.T., Belcher A.M. (2013). Tunable localized surface plasmon-enabled broadband light-harvesting enhancement for high-efficiency panchromatic dye-sensitized solar cells. Nano Lett.

[30] Si G.Y., Zhao Y.H., Lv J., Lu M., Wang F., Liu H., Xiang N., Huang T.J., Danner A.J., Teng J. (2013). Reflective plasmonic color filters based on lithographically patterned silver nanorod arrays. Nanoscale.

[31] Huang W., Qian W., Jain P.K., El-Sayed M.A. (2007). The effect of plasmon field on the coherent lattice phonon oscillation in electron-beam fabricated gold nanoparticle pairs. Nano Lett.

[32] Sun S., Leggett G.J. (2004). Matching the resolution of electron beam lithography by scanning near-field photolithography. Nano Lett.

[33] Atay T., Song J., Nurmikko A.V. (2004). Strongly interacting plasmon nanoparticle pairs: From dipole−dipole interaction to conductively coupled regime. Nano Lett.

[34] Si G.Y., Zhao Y.H., Lv J., Wang F., Liu H., Teng J., Liu Y.J. (2013). Direct and accurate patterning of plasmonic nanostructures with ultrasmall gaps. Nanoscale.

[35] Si G.Y., Zhao Y.H., Liu H., Teo S.L., Zhang M.S., Huang T.J., Danner A.J., Teng J.H. (2011). Annular aperture array based color filter. Appl. Phys. Lett.

[36] Jiang X., Gu Q., Wang F., Lv J., Ma Z., Si G. (2013). Fabrication of coaxial plasmonic crystals by focused ion beam milling and electron-beam lithography. Mater. Lett.

[37] Si G., Danner A.J., Teo S.L., Teo E.J., Teng J., Bettiol A.A. (2011). Photonic crystal structures with ultrahigh aspect ratio in lithium niobate fabricated by focused ion beam milling. J. Vac. Sci. Technol. B.

[38] Si G., Teo E.J., Bettiol A.A., Teng J., Danner A.J. (2010). Suspended slab and photonic crystal waveguides in lithium niobate. J. Vac. Sci. Technol. B.

[39] Liu Y.J., Si G.Y., Leong E.S.P., Xiang N., Danner A.J., Teng J.H. (2012). Light-driven plasmonic color filters by overlaying photoresponsive liquid crystals on gold annular aperture arrays. Adv. Mater.

[40] Liu Y.J., Si G.Y., Leong E.S.P., Wang B., Danner A.J., Yuan X.C., Teng J.H. (2012). Optically tunable plasmonic color filters. Appl. Phys. A.

[41] He J., Huang X., Li Y., Liu Y., Babu T., Aronova M.A., Wang S., Lu Z., Chen X., Nie Z. (2013). Self-assembly of amphiphilic plasmonic micelle-like nanoparticles in selective solvents. J. Am. Chem. Soc.

[42] Gandra N., Abbas A., Tian L., Singamaneni S. (2012). Plasmonic planet-satellite analogues: Hierarchical self-assembly of gold nanostructures. Nano Lett.

[43] Li X., Cole R.M., Milhano C.A., Bartlett P.N., Soares B.F., Baumberg J.J., de Groot C.H. (2009). The fabrication of plasmonic Au nanovoid trench arrays by guided self-assembly. Nanotechnology.

[44] Khoo I.C. (2009). Nonlinear optics of liquid crystalline materials. Phys. Rep.

[45] Zhao Y., Hao Q., Ma Y., Lu M., Zhang B., Lapsley M., Khoo I.C., Huang T.J. (2012). Light-driven tunable dual-band plasmonic absorber using liquid-crystal-coated asymmetric nanodisk array. Appl. Phy. Lett.

[46] Smalley J.S.T., Zhao Y., Nawaz A.A., Hao Q., Ma Y., Khoo I.C., Huang T.J. (2011). High contrast modulation of plasmonic signals using nanoscale dual-frequency liquid crystals. Opt. Express.

[47] Hao Q., Zhao Y., Juluri B.K., Kiraly B., Liou J., Khoo I.C., Huang T.J. (2011). Frequency-addressed tunable transmission in optically thin metallic nanohole arrays with dual-frequency liquid crystals. J. Appl. Phys.

[48] Liu Y.J., Leong E.S.P., Wang B., Teng J.H. (2011). Optical transmission enhancement and tuning by overlaying liquid crystals on a gold film with patterned nanoholes. Plasmonics.

[49] Liu Y.J., Ding X., Lin S.S., Shi J., Chiang I., Huang T.J. (2011). Surface acoustic wave driven light shutters using polymer-dispersed liquid crystals. Adv. Mater.

[50] Liu Y.J., Hao Q., Smalley J.S.T., Liou J., Khoo I.C., Huang T.J. (2010). A frequency-addressed plasmonic switch based on dual-frequency liquid crystal. Appl. Phy. Lett.

[51] Liu Y.J., Zheng Y.B., Shi J., Huang H., Walker T.R., Huang T.J. (2009). Optically switchable gratings based on azo-dye-doped, polymer-dispersed liquid crystals. Opt. Lett.

[52] Hsiao V.K.S., Zheng Y.B., Juluri B.K., Huang T.J. (2008). Light-driven plasmonic switches based on Au nanodisk arrays and photoresponsive liquid crystals. Adv. Mater.

[53] Chu K.C., Chao C.Y., Chen Y.F., Wu Y.C., Chen C.C. (2006). Electrically controlled surface plasmon resonance frequency of gold nanorods. Appl. Phys. Lett.

[54] Zografopoulos D.C., Beccherelli R. (2013). Long-range plasmonic directional coupler switches controlled by nematicliquid crystals. Opt. Express.

[55] Dickson W., Wurtz G.A., Evans P.R., Pollard R.J., Zayats A.V. (2008). Electronically controlled surface plasmon dispersion and optical transmission through metallic hole arrays using liquid crystal. Nano Lett.

[56] Kossyrev P.A., Yin A., Cloutier S.G., Cardimona D.A., Huang D., Alsing P.M., Xu J.M. (2005). Electric field tuning of plasmonic response of nanodot array in liquid crystal matrix. Nano Lett.

[57] De Sio L., Cunningham A., Verrina V., Tone C.M., Caputo R., Burgi T., Umeton C. (2012). Double active control of the plasmonic resonance of a gold nanoparticle array. Nanoscale.

[58] Khatua S., Chang W.S., Swanglap P., Olson J., Link S. (2011). Active modulation of nanorod plasmons. Nano Lett.

[59] Vivekchand S.R.C., Engel C.J., Lubin S.M., Blaber M.G., Zhou W., Suh J.Y., Schatz G.C., Odom T.W. (2012). Liquid plasmonics: Manipulating surface plasmon polaritons via phase transitions. Nano Lett.

[60] Zheng Y., Yang Y., Jensen L., Fang L., Juluri B.K., Flood A.H., Weiss P.S., Stoddart J.F., Huang T.J. (2009). Active molecular plasmonics: Controlling plasmon resonances with molecular switches. Nano Lett.

[61] Zheng Y., Hao Q., Wang Y., Kiraly B., Chiang I., Huang T.J. (2010). Light-driven artificial molucular machines. J. Nanophoton.

[62] Geandier G., Renault P., Bourhis E.L., Goudeau P., Faurie D., Bourlot C., Djemia P., Castelnau O., Cherif S.M. (2010). Elastic-strain distribution in metallic film-polymer substrate composites. Appl. Phy. Lett.

[63] Yang J., You J., Chen C., Hsu W., Tan H., Zhang X.W., Hong Z., Yang Y. (2011). Plasmonic polymer tandem solar cell. ACS Nano.

[64] Chah S., Noolandi J., Zare R.N. (2005). Undulatory delamination of thin polymer films on gold surfaces. J. Phys. Chem. B.

[65] Novo C., Funston A.M., Mulvaney P. (2008). Direct observation of chemical reactions on single gold nanocrystals using surface plasmon spectroscopy. Nat. Nanotechnol.

[66] Lioubimov V., Kolomenskii A., Mershin A., Nanopoulos D.V., Schuessler H.A. (2004). Effect of varying electric potential on surface-plasmon resonance sensing. Appl. Opt.

[67] Ung T., Liz-Marzan L.M., Mulvaney P. (1998). Controlled method for silica coating of silver colloids. Influence of coating on the rate of chemical reactions. Langmuir.

[68] Wu S.T. (1986). Birefringence dispersions of liquid crystals. Phys. Rev. A.

[69] Yang D.K., Wu S.T. (2006). Fundamentals of Liquid Crystal Devices.

[70] Khoo I.-C. (2007). Liquid Crystals.

[71] Li J., Gauza S., Wu S. (2004). Temperature effect on liquid crystal refractive indices. J. Appl. Phys.

[72] Zografopoulos D.C., Beccherelli R. (2013). Liquid-crystal-tunable metal–insulator–metal plasmonic waveguides and Bragg resonators. J. Opt.

[73] Schadt M. (1982). Dual-frequency addressing of field effects. Mol. Cryst. Liquid Cryst.

[74] Xianyu H., Wu S.T., Lin C.L. (2009). Dual frequency liquid crystals: A review. Liquid Cryst.

[75] Chang W., Lassiter J.B., Swanglap P., Sobhani H., Khatua S., Nordlander P., Halas N.J., Link S. (2012). A plasmonic Fano switch. Nano. Lett.

[76] Liu Y.J., Dai H.T., Sun X.W. (2011). Holographic fabrication of azo-dye-functionalized photonic structures. J. Mater. Chem.

[77] Liu Y.J., Su Y.-C., Hsu Y.-J., Hsiao V.K.S. (2012). Light-induced spectral shifting generated from azo-dye doped holographic 2D gratings. J. Mater. Chem.

[78] De Sio L., Tedesco A., Tabirian N., Umeton C. (2010). Optically controlled holographic beam splitter. Appl. Phys. Lett.

[79] De Sio L., Serak S., Tabirian N., Umeton C. (2011). Mesogenic versus non-mesogenic azo dye confined in a soft-matter template for realization of optically switchable diffraction gratings. J. Mater. Chem.

[80] Liu Y.J., Cai Z.Y., Leong E.S.P., Zhao X.S., Teng J.H. (2012). Optically switchable photonic crystals based on inverse opals partially infiltrated by photoresponsive liquid crystals. J. Mater. Chem.

[81] Liu Y.J., Dai H.T., Leong E.S.P., Teng J.H., Sun X.W. (2012). Azo-dye-doped absorbing photonic crystals with purely imaginary refractive index contrast and all-optically switchable diffraction properties. Opt. Mater. Express.

[82] De Sio L., Klein G., Serak S., Tabiryan N., Cunningham A., Tone C.M., Ciuchi F., Bürgi T., Umeton C., Bunning T. (2013). All-optical control of localized plasmonic resonance realized by photoalignment of liquid crystals. J. Mater. Chem. C.

[83] Liu Y.J., Zheng Y.B., Liou J., Chiang I.-K., Khoo I.C., Huang T.J. (2011). All-optical modulation of localized surface plasmon coupling in a hybrid system composed of photo-switchable gratings and Au nanodisk arrays. J. Phys. Chem. C.

[84] Tamai N., Miyasaka H. (2000). Ultrafast dynamics of photochromic systems. Chem. Rev.

[85] De Sio L., Ricciardi L., Serak S., La Deda M., Tabiryan N., Umeton C. (2012). Photo-sensitive liquid crystals for optically controlled diffraction gratings. J. Mater. Chem.

[86] Hrozhyk U.A., Serak S.V., Tabiryan N.V., Hoke L., Steeves D.M., Kimball B.R. (2010). Azobenzene liquid crystalline materials for efficient optical switching with pulsed and/or continuous wave laser beams. Opt. Express.

[87] Hrozhyk U.A., Serak S.V., Tabiryan N.V., Hoke L., Steeves D.M., Kimball B., Kedziora G. (2008). Systematic study of absorption spectra of donor–acceptor azobenzene mesogenic structures. Mol. Cryst. Liquid Cryst.

[88] Li J., Gauzia S., Wu S.T. (2004). High temperature-gradient refractive index liquid crystals. Opt. Express.

[89] Li J., Wen C.H., Gauza S., Lu R., Wu S.T. (2005). Refractive indices of liquid crystals for display applications. J. Display Technol.

[90] Cetin A.E., Mertiri A., Huang M., Erramilli S., Altug H. (2013). Thermal tuning of surface plasmon polaritons using liquid crystals. Adv. Opt. Mater.

[91] Bardhan R., Lal S., Joshi A., Halas N.J. (2011). Theranostic nanoshells: From probe design to imaging and treatment of cancer. Acc. Chem. Res.

[92] De Sio L., Placido T., Serak S., Comparelli R., Tamborra M., Tabiryan N., Curri M.L., Bartolino R., Umeton C., Bunning T. (2013). Nano-localized heating source for photonics and plasmonics. Adv. Opt. Mater.

[93] Richardson H.H., Hickman Z.N., Gocorov A.O., Thomas A.C., Zhang W., Kordesch M.E. (2006). Thermooptical properties of gold nanoparticles embedded in ice:  Characterization of heat generation and melting. Nano Lett.

[94] Wilson O.M., Hu X., Cahill D.G., Braun P.V. (2002). Colloidal metal particles as probes of nanoscale thermal transport in fluids. Phys. Rev. B.

[95] Khatua S., Manna P., Chang W.-S., Tcherniak A., Friedlander E., Zubarev E.R., Link S. (2010). Plasmonic nanoparticles-liquid crystal composites. J. Phys. Chem. C.

[96] Liu Q.K., Cui Y.X., Gardner D., Li X., He S.L., Smalyukh I.I. (2010). Self-alignment of plasmonic gold nanorods in reconfigurable anisotropic fluids for tunable bulk metamaterial applications. Nano Lett.

[97] Umadevi S., Feng X., Hegmann T. (2013). Large area self-assembly of nematic liquid-crystal-functionalized gold nanorods. Adv. Funct. Mater.

[98] Milette J., Cowling S.J., Toader V., Lavigne C., Saez I.M., Bruce Lennox R., Goodby J.W., Reven L. (2012). Reversible long range network formation in gold nanoparticle-nematic liquid crystal composites. Soft Matter.

[99] Wu B.-G., Erdmann J.H., Doane J.W. (1989). Response times and voltages for PDLC light shutters. Liq. Cryst.

[100] Liu Y.J., Sun X.W. (2007). Electrically switchable computer-generated hologram recorded in polymer-dispersed liquid crystals. Appl. Phys. Lett.

[101] Tondiglia V.P., Natarajan L.V., Sutherland R.L., Bunning T.J., Adams W.W. (1995). Volume holographic image storage and electro-optical readout in a polymer-dispersed liquid-crystal film. Opt. Lett.

[102] Liu Y.J., Sun X.W., Liu J.H., Dai H.T., Xu K.S. (2005). A polarization insensitive 2 × 2 optical switch fabricated by liquid cystal-polymer composite. Appl. Phys. Lett.

[103] Liu Y.J., Sun X.W., Dai H.T., Liu J.H., Xu K.S. (2005). Effect of surfactant on the electro-optical properties of holographic polymer dispersed liquid crystal Bragg gratings. Opt. Mater.

[104] Sun J., Wu S.T. (2014). Recent advances in polymer network liquid crystal spatial light modulators. J. Polym. Sci. Part B Polym. Phys.

[105] Yan J., Rao L., Jiao M., Li Y., Cheng H.C., Wu S.T. (2011). Polymer-stabilized optically isotropic liquid crystals for next-generation display and photonics applications. J. Mater. Chem.

[106] Gauza S., Wang H., Wen C.H., Wu S.T., Seed A.J., Dabrowski R. (2003). High birefringence isothiocyanato tolane liquid crystals. Jpn. J. Appl. Phys.

[107] Dabrowski R., Kula P., Herman J. (2013). High birefringence liquid crystals. Crystals.

[108] Arakawa Y., Kang S., Nakajima S., Sakajiri K., Cho Y., Kawauchi S., Watanabe J., Konishi G. (2013). Diphenyltriacetylenes: Novel nematic liquid crystal materials and analysis of their nematic phase-transition and birefringence behaviours. J. Mater. Chem. C.

[109] Gauza S., Wen C.H., Wu S.T., Janarthanan N., Hsu C.S. (2004). Super high birefringence isothiocyanato biphenyl-bistolane liquid crystals. Jpn. J. Appl. Phys.

[110] De Sio L., Umeton C. (2010). Dual-mode control of light by two-dimensional periodic structures realized in liquid-crystalline composite materials. Opt. Lett.

